# Performance of Impregnated Paper Decorated Blockboard Manufactured Using HDF as Equilibrium Layer

**DOI:** 10.3390/ma15186342

**Published:** 2022-09-13

**Authors:** Lu Fang, Xizhen Lu, Xianfeng Mo, Xinhao Zhang, Chengsheng Gui

**Affiliations:** 1 Co-Innovation Center of Efficient Processing and Utilization of Forest Resources, Nanjing Forestry University, Nanjing 210037, China; 2 College of Furnishings and Industrial Design, Nanjing Forestry University, Nanjing 210037, China; 3 Zhejiang Shenghua Yunfeng Greeneo Co., Ltd., Huzhou 313200, China

**Keywords:** impregnated paper, blockboard, equilibrium layer, surface cracking resistance

## Abstract

In order to further improve the performance of impregnated paper decorated blockboard (ecological board), high-density fiberboard (HDF) was selected as the equilibrium layer to replace the commonly used poplar veneer. Results showed that the performance of HDF ecological board can be comparable to that of poplar veneer ecological board. It had good appearance quality, and its surface scratch resistance, surface wear resistance, water resistance and mechanical properties met the requirements of National Standard GB/T 34722-2017. The surface cracking resistance of the ecological board prepared with HDF as the equilibrium layer reached the highest level (grade 5), far better than that of the poplar veneer ecological board. This was because HDF was a relatively homogeneous material, and its dry shrinkage in both the transverse direction and along the grain direction was much lower than that of the poplar veneer. This characteristic of HDF made it possible to improve the dimensional stability and bending resistance of blockboard substrates under dry and hot conditions. The formaldehyde emission of the HDF ecological board was higher than that of the poplar veneer ecological board, but it met the formaldehyde emission requirements of indoor materials according to GB 18580-2001.

## 1. Introduction

**Figure 1:**
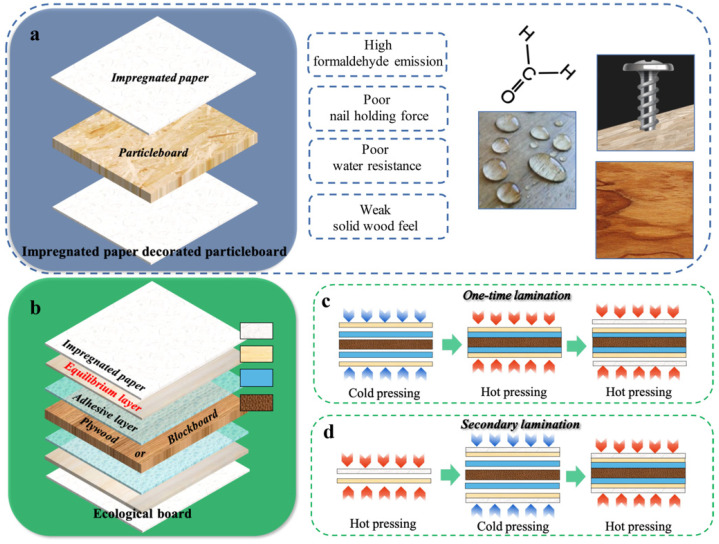
Impregnated paper decorated wood-based panels. (**a**) Structure of impregnated paper decorated particleboard; (**b**) Structure of ecological board; (**c**) One-time lamination method; (**d**) Secondary lamination method.

Customized furniture has developed rapidly in recent years because of many advantages, such as personalized design, high space utilization, fashionable appearance, and so on [1,2,3]. The raw materials for manufacturing customized furniture were mainly decorated wood-based panels. Commonly used decorative materials mainly included wood veneer, textile fabric, plastic film, coatings, and impregnated paper [4,5,6,7,8], among which resin impregnated paper was the most popular, accounting for 60~70% of all the decorative materials in the wood industry [9,10,11]. Among various wood-based panels, particleboard has become the preferred substrate due to its good processability, low price as well as isotropy [12,13,14,15]. The structure of impregnated paper decorated particleboard is shown in Figure 1a. At present, particleboard decorated with impregnated paper has been selected by many furniture enterprises as the main materials for the production of the customized cabinets and customized wardrobes [16,17]. However, with the increase of market demand and quality requirements, the defects of this kind of panel, such as high formaldehyde emission, weak nail holding force, poor water resistance, and inferior solid wood feelings, have gradually attracted people’s attention and limited its further application [18,19,20]. The International Agency for Research on Cancer reclassified formaldehyde from “probably carcinogenic to humans” to “carcinogenic to humans” in 2004. The European Union, the USA, China, and Japan now have legislation regulating the allowed levels of formaldehyde emissions from wood-based products [21]. Zhou et al. [22] found that the strength of impregnated paper decorated particleboard decreased with the temperature, and the formaldehyde emissions of the panels sharply increased when the temperature was higher than 60 °C. Some scholars tried to replace the particleboard substrate with plywood or blockboard. In recent years, a unique decorative wood-based panel called ecological board was eventually developed in China [23,24,25]. Peng [26] systematically introduced the raw materials, production methods, and hot-pressing process parameters of ecological board, and analyzed the causes of relevant defects, such as delamination, bubbling, dry and wet flowers, ripple and cracking on the surface of the decorative board. Different from the particleboard substrate, the surface quality of the blockboard or plywood substrate was usually not good in order to reduce the panel cost. Therefore, it was necessary to add an equilibrium layer between the impregnated paper and the substrate when preparing ecological board [27]. As shown in Figure 1b, the existence of this equilibrium layer made the structure of the decorative panel much more complex. It is of great significance to explore the effects of equilibrium layer on the physical and mechanical properties of impregnated paper decorated wood-based panel.

The equilibrium layer was usually a wood veneer with a thickness of 0.5 to 1 mm. Commonly used wood included poplar, beech, or reconstituted decorative veneer. The addition of this equilibrium layer can not only repair the defects on the substrate, but also acted as a buffer material to protect the substrate in the subsequent hot-pressing process. The equilibrium layer may be composited with the substrate or impregnated paper in advance. The method of using impregnated paper to decorate the equilibrium layer/substrate composite was called one-time lamination (Figure 1c), which was also the most commonly used preparation process for ecological board at present. On the other hand, if the impregnated paper was used for substrate decoration after pre compounding with the equilibrium layer, this method of preparing the ecological board was defined as secondary lamination (Figure 1d). Generally, the ecological board prepared by one-time lamination method had lower formaldehyde emission and higher surface brightness, and relatively low limit on the equilibrium layer. Lu et al. [25] found that the composite method of equilibrium layer has little effect on the surface wear resistance, surface scratch resistance, and water resistance of the ecological board. The mechanical properties were also less affected. However, the ecological board prepared by the one-time lamination method has poor ability to resist the change of external temperature and humidity [28]. This was because the impregnated paper was only hot-pressed once in this process, and the resins in the impregnated paper cannot be sufficiently cured in such a short time. A large number of unreacted hydroxyl groups remaining in the uncured melamine resin easily formed hydrogen bonds with water, resulting in uneven stress and cracks on the impregnated paper surface. This issue can be well solved by using the secondary lamination method, but the cleanliness of the ecological board surface was significantly reduced, and the carbonization of impregnated paper occurred very easily. The anisotropy of the equilibrium layer and the substrate (blockboard or plywood) was also an important reason for the surface cracking of ecological board. Some scholars tried to improve the cracking resistance by adding a layer of isotropic materials, such as non-woven fabric, kraft paper, on the substrate surface [29,30]. These methods have a certain effect on suppressing the uneven deformation of the panel, but the preparation process will become more complicated, and the manufacturing cost will be higher.

In this paper, homogeneous high-density fiberboard (HDF) was selected as an alternative equilibrium layer to manufacture impregnated paper decorated blockboard in order to alleviate the cracking of ecological board. The surface quality, water resistance, mechanical properties and formaldehyde emission of the decorative panel were systematically analyzed and compared with the commonly used impregnated paper decorated blockboard with poplar veneer as an equilibrium layer.

## 2. Materials and Methods

### 2.1. Materials

Chinese fir core eucalyptus blockboard with a dimension of 300 mm × 300 mm × 16.2 mm and moisture content of 8~10% was used as the substrate panel. The decorative paper used in this study was a milk white design with quantitative of 102.7 g m^−2^. Its pre-curing degree and formaldehyde emission was 46.7% and 0.6 mg/L, respectively. E1 grade HDF was selected as the equilibrium layer to prepare ecological board, with a moisture content of 5~8% and a density of 0.82 g cm^3^. Its dimension was 300 mm × 300 mm × 0.8 mm. In addition, poplar veneer (*P. euramericana cv.*) with a moisture content of 5~8% was selected as a control equilibrium layer material. Its dimension was 300 mm × 300 mm × 0.7 mm. Commercially available liquid UF resins were used as adhesives. The liquid UF resins had a solids content of 53.3%, a pH of about 7.6 and a viscosity of 90 mPa·s. 1% NH_4_Cl based on the weight of the UF resin liquid was used as a hardener. The NH_4_Cl was prepared into 20% aqueous solution and added to the UF resin liquid. All materials in this paper were kindly supplied by Zhejiang Shenghua Yunfeng Greeneo Co., Ltd., Huzhou, China.

### 2.2. Production of Impregnated Paper Decorated Blockboard

As shown in Figure 1c, the blockboard substrate was first composited with the equilibrium layer using UF resin as adhesives. The UF resin content was 140 g m^−2^ based on the weight of the liquid in single glue line. The assembled equilibrium layer/blockboard composite was cold pressed at 0.55 MPa for 1 h at first, and then hot pressed at 120 °C and 0.65 MPa for 390 s. Before decoration with the impregnated paper, the equilibrium layer/blockboard composites was placed at room temperature for 24 h. In the finishing step, the hot-pressing temperature, hot-pressing pressure, and hot-pressing times were 125 °C, 0.65 MPa and 320 s, respectively.

### 2.3. Composites Characterization

#### 2.3.1. Appearance Quality

The appearance quality, including blisters, bulge, surface ripples, surface waviness, dry flowers, wet flowers, on the surface of impregnated decorated blockboard was observed according to the standard of “surface decorated plywood and blockboard with paper impregnated thermosetting resins” (GB/T 34722-2017). The surface cleanliness was also recorded.

#### 2.3.2. Surface Properties

The surface cracking resistance, surface wear resistance and surface scratch resistance of the impregnated decorated blockboard were tested according to the standard of “Test methods of evaluating the properties of wood-based panels and surface decorated wood-based panels” (GB/T 17657-2013). For the surface cracking resistance test, the specimen was heated in an oven at 70 °C for 24 h at first, then equilibrated at 25 °C and 50% RH, and finally the surface changes were recorded with a 6× magnifying glass at an illuminance of 800~1000 lx. The surface wear resistance test was performed in Taber 5135, which was operated at 350 rpm with a load of 4.9 N. The weight loss on the flat surface of the specimen (100 mm × 100 mm) was recorded. The surface of the specimen was scored with a diamond needle to evaluate its scratch resistance. During the test, the weight was fixed at 1.5 N.

#### 2.3.3. Physical-Mechanical Properties

Surface bonding strength and three-point static bending strength of the impregnated paper decorated blockboard were measured using a Multi-Function Mechanical Testing machine (CMT4204, Sansi Co., Ltd., Shenzhen, China). The maximum failure load value was output to calculate the results. Some details of the test method were in the Supplementary Material File S1.

Immersion peeling performance and thickness swelling (TS) of the impregnated paper decorated blockboard were also tested. For the immersion peel test, the specimen was first immersed in water at 63 °C for 3 h, then put into the oven at 63 °C for 3 h, and the peeling length was recorded. TS was calculated from the difference in specimen thickness before and after soaking in water for 24 h.

All specimens for the physical-mechanical test were prepared according to the standard GB/T 17657-2013. Before testing, all specimens were conditioned at 25 °C and 65% humidity for at least 24 h. At least four samples were tested for each condition, and the test results were their average values.

#### 2.3.4. Formaldehyde Emission

The impregnated decorated blockboard with a size of 150 mm × 50 mm and 300 mL distilled water were put into the desiccator at 20 °C for 24 h. The formaldehyde emission of the sample was obtained by analyzing the formaldehyde concentration in distilled water according to the desiccator method in GB/T 17657-2013. The test result was the average value of formaldehyde content of the two test pieces.

#### 2.3.5. Scanning Electron Microscopy (SEM)

The impregnated paper decorated blockboard was cut into 5 mm × 5 mm and sprayed with gold. The bonding interface between impregnant paper and equilibrium layer, and the bonding interface between equilibrium layer and blockboard substrate were observed by a Quanta-200 ESEM (Hillsboro, OR, USA).

## 3. Results and Discussion

### 3.1. Surface Properties of HDF Ecological Board

Ecological board is mainly used to manufacture various furniture products. The surface quality of ecological board directly determined the quality grade and category of furniture products. The appearance quality, surface cracking resistance, surface wear resistance and surface scratch resistance were used to evaluate the surface quality of ecological boards in this study.

#### 3.1.1. Surface Cracking Resistance

Surface cracking resistance is an effective method to measure the adaptability of ecological board under the rapid change of environment, such as temperature and humidity. In recent years, the quality of ecological boards has steadily improved except for the surface cracking resistance. Poor surface cracking resistance has become one of the most important factors hindering the replacement of impregnated paper decorated particleboard by ecological board.

##### Surface Cracking Resistance Grade

According to the requirements in the national standard GB/T 34722-2017, the surface cracking resistance grade shall be higher than 4. In other words, only a single tiny crack was allowed on the board surface under a 6× magnifying glass after the cracking test. Previous studies showed that the more obvious the anisotropy of the equilibrium layer between the substrate and the impregnated paper, the lower the cracking resistance grade [28]. As shown in Table 1, various cracks appeared on the whole surface of the most commonly used poplar veneer ecological board after the cracking test was finished. The surface cracking resistance grade can only reach grade 1. When HDF was selected as the equilibrium layer, the situation was completely different. There were no cracks on both sides of the HDF ecological board, and the cracking resistance grade reached the highest level (grade 5). This was mainly caused by the different properties of HDF and poplar veneer.

##### Performance of HDF under Dry Heat Conditions

In order to further analyze the influence mechanism of surface cracking resistance of ecological boards, HDF, poplar veneer, blockboard, as well as the equilibrium layer/blockboard composites were treated at 70 °C for 24 h, respectively, (dry heat condition for surface cracking experiment). The change of size and strength of each material after dry heat treatment was recorded.

**Figure 2:**
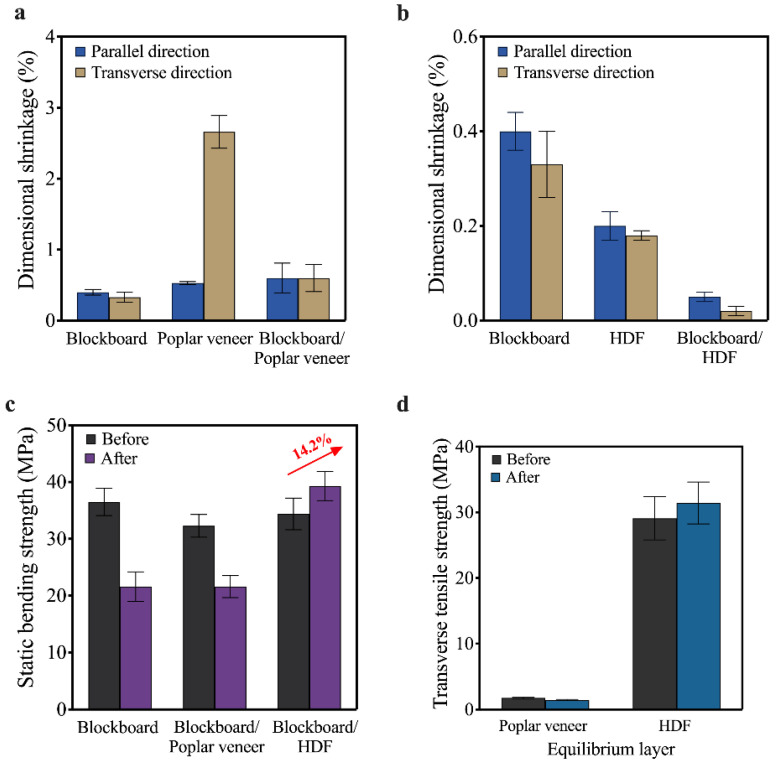
Effect of dry heat treatment on the equilibrium layer and its composites: (**a**) dimensional shrinkage of poplar veneer, blockboard and poplar/blockboard composite; (**b**) dimensional shrinkage of HDF, blockboard and HDF/blockboard composite; (**c**) static bending strength of equilibrium layer/substrate composites; and (**d**) transverse tensile strength of different equilibrium layers.

HDF is a relatively uniform material with little difference in dimensional shrinkage in all directions [31]. As shown in Figure 2a,b, the dimensional shrinkage of HDF in the transverse direction and along the grain direction were 0.2% and 0.18%, respectively, which were far lower than not only the poplar veneer, but also the blockboard substrate. Therefore, when the blockboard substrate was composited with HDF, its ability to resist dry heat change can be significantly improved. As shown in Figure 2b, the dry shrinkage of the HDF/blockboard composites along the grain and across the grain decreased from 0.40% and 0.33% to 0.05% and 0.02%, respectively. On the contrary, the size of poplar veneer after dry heat treatment varied greatly due to the heterogeneity of wood. The transverse grain shrinkage of poplar veneer reached 2.66%, which was about 15 times of HDF and 8 times of blockboard substrate. This in turn led to a higher dimensional shrinkage of blockboard/poplar veneer composite than the blockboard substrate.

The strength of the HDF/substrate composite was not affected by the dry heat treatment due to the strengthening effect of HDF on the dimensional stability of blockboard substrate. As shown in Figure 2c, the bending strength of the composite increased from 34.4 MPa to 39.3 MPa due to the short-time heat treatment. For the same reason, the static bending strength of blockboard and poplar veneer/blockboard composites deteriorated after the dry heat treatment. Results showed that the retention rate was 59.2% for the substrate and 66.9% for the poplar veneer/blockboard composites. The excellent tensile strength of HDF also played an important role in the improvement of surface cracking resistance. As shown in Figure 2d, the tensile strength of HDF and poplar veneer were around 29.2 MPa and 1.8 MPa, respectively. However, the tensile strength of both materials was little-affected by the dry heat treatment since the treatment temperature was lower than 100 °C [32].

##### Interface Morphology

**Figure 3:**
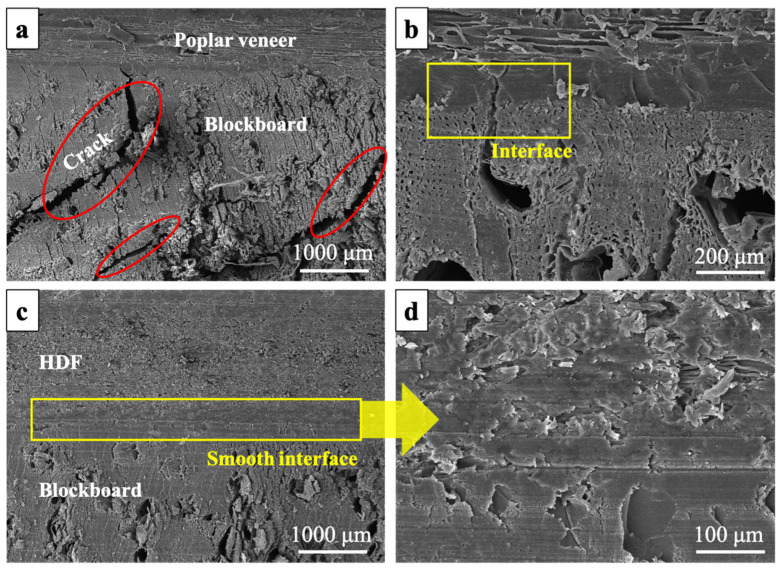
Interface morphology of the ecological board after dry heat treatment: (**a**) interface of poplar veneer/blockboard (100×); (**b**) interface of poplar veneer/blockboard (500×); (**c**) interface of HDF/blockboard (50×); and (**d**) interface of HDF/blockboard (500×).

The remarkable difference in dimensional stability and mechanical strength between HDF and poplar veneer made the thermal stress generated in the two kinds of ecological boards different during the cracking test. Figure 3 showed the interface morphology of the ecological board after dry heat treatment. As shown in Figure 3a,b, when poplar veneer was used as equilibrium layer, a large number of fragments and obvious cracks were found on the veneer, blockboard as well as their interfaces. The impregnated paper on the surface of the ecological board cracked because it could not bear these stresses. When was used as the equilibrium layer, the situation was totally different for HDF ecological board. As shown in Figure 3c,d, the interface of the ecological board was still smooth after the dry heat treatment.

#### 3.1.2. Other Surface Properties of HDF Ecological Board

In addition to the surface cracking performance, the appearance quality, surface wear resistance, and surface scratch resistance of HDF ecological board were also the focus of attention for researchers. Results showed that the overall appearance quality of the ecological board prepared with HDF as equilibrium layer was good. No contamination, ripple, bubble, dry flower, and wet flower were found on the surface of HDF ecological board. The surface scratch resistance of HDF ecological board met the requirements in GB/T 34722-2017. When the diamond needle was used to scratch the surface, no continuous scratch greater than 90% occurred on the HDF ecological board. Surface wear resistance is an important parameter to characterize the performance of the decorative layer on the surface of ecological board under certain friction conditions. As shown in Table 2, the surface wear resistance of HDF board was comparable to that of poplar ecological board. The wear values of ecological boards prepared with different equilibrium layers were less than 80 mg/100 r, and there was no bottom exposure after the wear test.

### 3.2. Physical-Mechanical Properties of HDF Ecological Board

#### 3.2.1. Water Resistance

**Figure 4:**
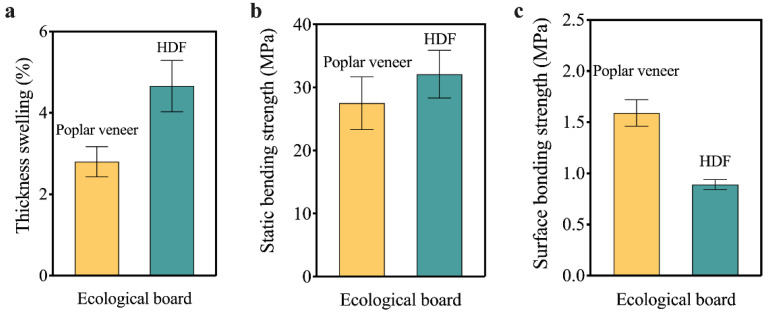
Physical-mechanical properties of the impregnated paper decorated blockboard prepared with the two equilibrium layers: (**a**) 24 h thickness swelling rate; (**b**) static bending strength; and (**c**) surface bonding strength.

The water resistance determined the place where the ecological board can be applied. Since HDF was much easier to absorb water than poplar veneer, the water resistance of HDF ecological board must be considered. For decorative panels, the immersion peeling performance is an important index to evaluate their water resistance. In this paper, a type II water resistance test (immersed in water at 63 °C for 3 h, and then put into the oven at 63 °C for 3 h) was carried out. The results showed that there was no delamination at each adhesive layer, which means that HDF ecological board can be used as an indoor material. However, the 24-h thickness swelling rate of HDF ecological board was 4.66%, 66% higher than that of poplar ecological board (Figure 4a).

#### 3.2.2. Mechanical Properties

Mechanical properties of the impregnated paper decorated blockboard prepared with the two equilibrium layers were shown in Figure 4b,c. Due to the higher static bending strength of blockboard substrate/HDF composite (Figure 2c), the static bending strength of HDF ecological board was higher than that of poplar ecological board (Figure 4b). However, the difference between them was not significant.

**Figure 5:**
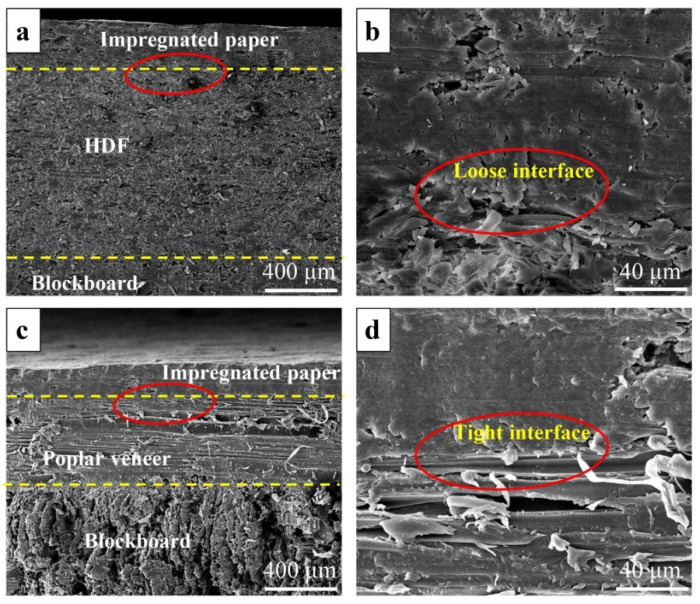
Interface morphology between impregnated paper and different equilibrium layers: (**a**) interface between impregnated paper and HDF (100×); (**b**) interface between impregnated paper and HDF (1000×); (**c**) interface between impregnated paper and poplar veneer (100×); and (**d**) interface between impregnated paper and poplar veneer (1000×).

**Figure 6:**
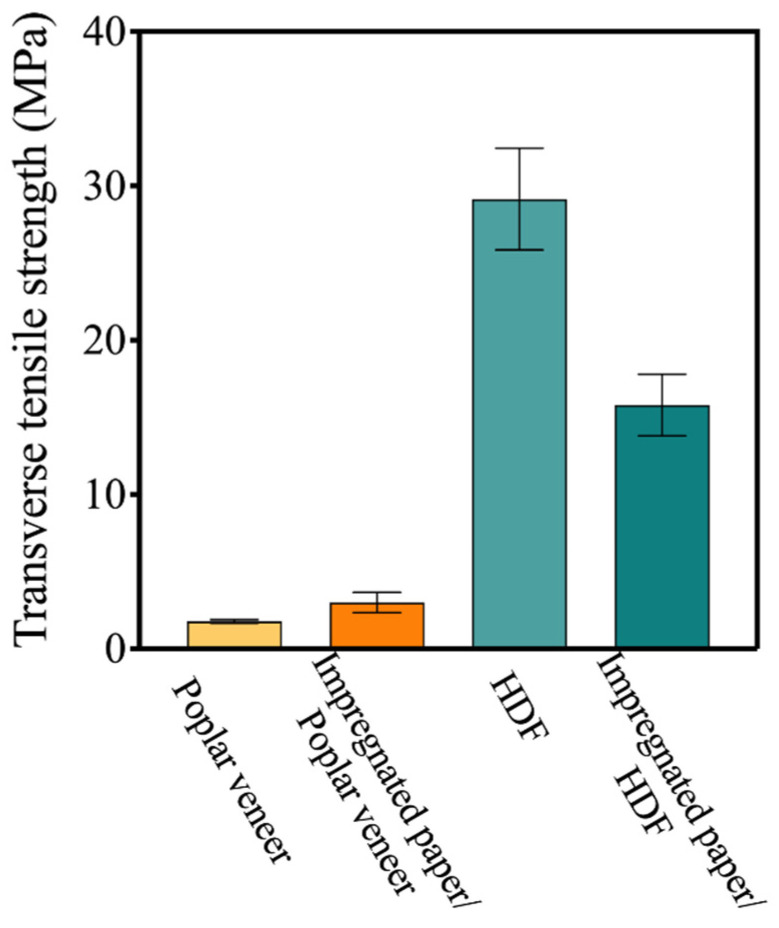
Transverse tensile strength of impregnated paper/equilibrium layer composites.

The surface bonding strength can be used to evaluate the bonding quality between the decorative paper and the substrate. According to the National Standard GB/T 34722-2017, the surface bonding strength of the ecological board shall not be less than 0.6 MPa. As shown in Figure 4c, the surface bonding strength of HDF ecological board was 0.89 MPa, which indicated that a relatively good bonding interface was formed between impregnated paper, HDF and blockboard substrate during the preparation of ecological board. However, it was lower than the poplar veneer ecological board. This was because melamine adhesive in the impregnated paper was much easier to penetrate the porous structure of the poplar veneer, so impregnated paper can form a much more ideal bonding structure with the poplar veneer [33]. As shown in Figure 5a, the interface between impregnated paper and HDF was relatively loose, and interface defects can be observed at 1000 times magnification (Figure 5b). Due to the interface effect [34], when HDF was composited with the impregnated paper, its tensile strength decreased by 100%. On the other hand, when poplar veneer was composited with the impregnated paper, its tensile strength was improved (Figure 6), which was also due to the tight bonding interface between the impregnated paper and the poplar veneer.

### 3.3. Effect of Equilibrium Layer on the Formaldehyde Emission of Ecological Board

**Figure 7:**
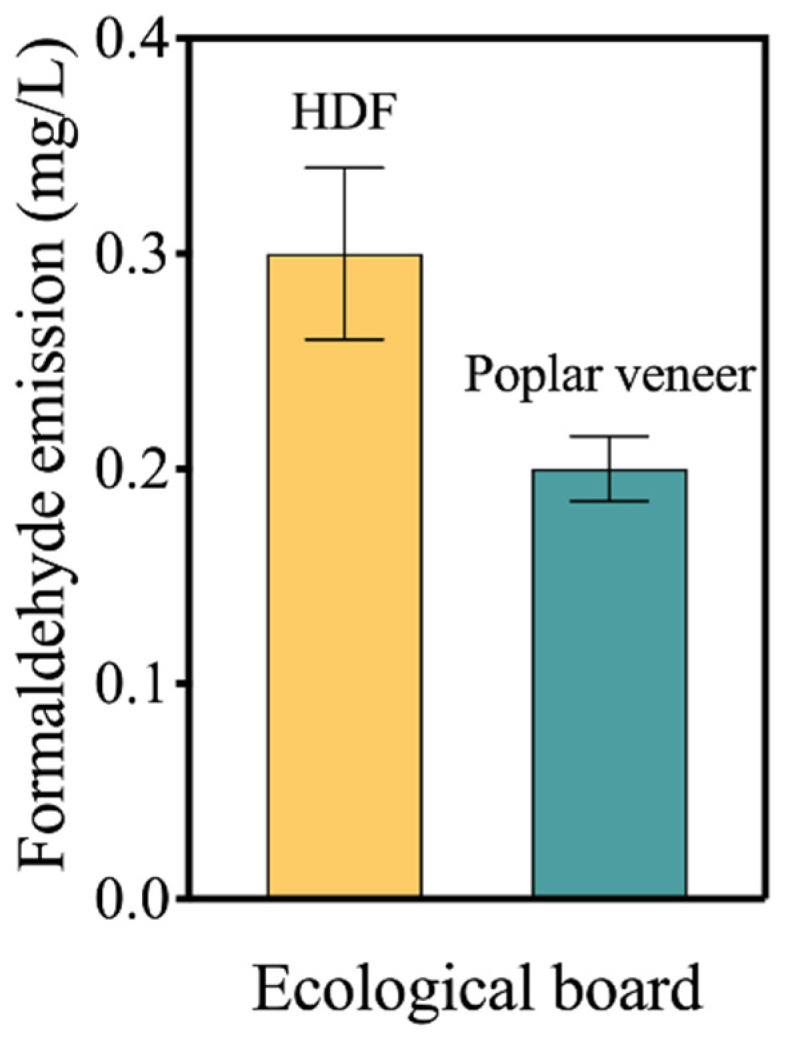
Formaldehyde emission of the impregnated paper decorated blockboard prepared with the two equilibrium layers.

Although impregnated paper can be used as a barrier to hinder the outward release of formaldehyde, the formaldehyde problem still deserves attention due to increasingly stringent policies. According to Standard GB 18580-2001 (“Indoor decorating and refurbishing materials-formaldehyde release limit of wood-based decorative materials and their products”), when decorative wood-based panels are directly used indoors, their formaldehyde emission shall not be higher than 1.5 mg/L. As shown in Figure 7, the formaldehyde emission of HDF ecological board was 0.3 mg/L, meeting the formaldehyde limit requirements. However, it was 50% higher than that of the poplar veneer ecological board. This is because a large amount of adhesive was used in the preparation of HDF, which was the main source of formaldehyde.

## 4. Conclusions

Ecological board has attracted extensive attention in the field of customized furniture. In the structure of ecological board, the equilibrium layer can not only increase the surface flatness of the blockboard substrate, but also protect and buffer the substrate. Wood veneer is the most commonly used equilibrium layer, but the cracking resistance grade of wood veneer ecological board can only reach grade 1, which was due to the anisotropy and obvious shrinkage under dry and hot conditions. This problem can be well solved when homogeneous high-density fiberboard (HDF) is selected as an alternative material for wood veneer. Results showed that the cracking resistance grade of HDF ecological board can reach the highest level. In addition, HDF ecological board has a similar appearance quality and physical-mechanical properties as the poplar veneer ecological board. HDF does not have the advantage of a solid wood feeling, but it is more adaptable to temperature and humidity changes. Therefore, when the ecological board needs to be exported to different regions for use, it is an economic and effective method to choose HDF as the equilibrium layer. On the contrary, wood veneer ecological board has more stringent requirements on the use environment.

## Figures and Tables

**Table 1:** Surface cracking resistance of ecological board prepared with different equilibrium layers.

Equilibrium Layer	Specimen	Surface Cracks on Both Sides	Surface Topography
HDF	1	no crack	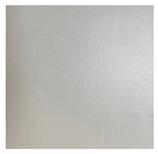
	2	no crack	
	3	no crack	
	4	no crack	
Poplar veneer	5	10–20 cracks, 5–200 mm for each crack	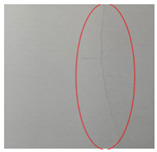
	6	5–10 cracks, 5–150 mm for each crack	
	7	more than 20 cracks, 50–200 mm for each crack	
	8	0–5 cracks, 5–100 mm for each crack	

**Table 2:** Surface wear resistance of ecological board prepared with different equilibrium layers.

Equilibrium Layer	Wear Revolutions (R)	Wear Values (mg/100 r)	Surface Wear Condition
HDF	350	61	No bottom exposure
Poplar veneer	350	55	No bottom exposure

## Data Availability

The data presented in this study are available on request from the corresponding author.

## Supplementary Materials

The following supporting information can be downloaded at: https://www.mdpi.com/article/10.3390/ma15186342/s1, File S1: Details of surface bonding strength.

## References

[1] Mikulskienė B., Moskvina J. (2020). Peculiar properties of the pricing process in customized furniture manufacturing enterprises. J. Revenue Pricing Manag..

[2] Lihra T., Buehlmann U., Graf R. (2012). Customer preferences for customized household furniture. J. For. Econ..

[3] Ren J., Xiong X.Q., Zhao J.Z., Fei J.L., Zhang J. (2022). Research on standardized design of solid wood customized cabinet doors. J. For. Eng..

[4] Wang R., Xu D.L., Yang Y., Yan W.W., Zhan X.X., Xu X.W. (2020). Industrial resin-impregnation and overlaying application of reconstituted decorative veneers. J. For. Eng..

[5] Zhang X.H., Fang L., Wu Z.H., Zhao J.Z. (2021). Preparation and properties of EVA film reinforced decorative veneer. J. For. Eng..

[6] Kandelbauer A., Petek P., Medved S., Pizzi A., Teischinger A. (2010). On the performance of a melamine–urea–formaldehyde resin for decorative paper coatings. Eur. J. Wood Wood Prod..

[7] Chang L., Luo S.P., Gao L., Ren Y.P., Tang Q.H., Chen Y.P. (2022). Composite waterborne polyurethane reinforced with silane modified lignin as an adhesive between polypropylene decorative films and wood-based panels. Polym. Eng. Sci..

[8] Xiong X.Q., Niu Y.T., Yuan Y.Y., Zhang L.T. (2020). Study on Dimensional Stability of Veneer Rice Straw Particleboard. Coatings.

[9] Wang W.L., Peng J.D., Zhao Z.Y., Zhou X.J., Zhang J., Du G.B. (2020). Modification of melamine formaldehyde resin for decoration board. J. For. Eng..

[10] Enzensberger W. (1961). On the surface finishing of particleboard with resin-impregnated paper layers. Eur. J. Wood Wood Prod..

[11] Wang R., Lv B. (2019). Analysis and consideration of wooden household surface decoration industry in China. China Wood-Based Panels.

[12] Zhang J.Z., Li J.Z., Zhang S.F. (2011). Properties of Particleboard Manufactured with Modified Urea-Formaldehyde Resin. Adv. Mater. Res..

[13] Liu M., Shen J., Wang W.D., Xv W., Wang H.Y. (2021). Analysis of very volatile organic compounds (VVOC) and odor emission from decorative particleboard. J. Beijing For. Univ..

[14] Niemz P., Sandberg D. (2022). Critical wood-particle properties in the production of particleboard. Wood Mater. Sci. Eng..

[15] Nemli G., Ors Y., Kalaycioglu H. (2005). The choosing of suitable decorative surface coating material types for interior end use applications of particleboard. Constr. Build. Mater..

[16] Ren J., Xiong X.Q. (2022). Digital Design Process and Part Family Division of Solid Wood Custom Cabinet Door Based on Multi-attribute Overlapping Clustering Technology. BioResources.

[17] Niu Y.T., Xiong X.Q. (2022). Investigation on Panel Material Picking Technology for Furniture in Automated Raw Material Warehouses. BioResources.

[18] Tian J., Qin Y.X., Yang S.J., Lv Y.T., Huang H.M., Ye D.Q. (2022). Traceability and Emission Characteristics Analysis of VOCs from Typical Plates in Customized Furniture. China Environ. Sci..

[19] Xiong X.Q., Ma Q.R., Yuan Y.Y., Wu Z.H., Zhang M. (2020). Current situation and key manufacturing considerations of green furniture in China A review. J. Clean. Prod..

[20] Nemli G., Colakoglu G. (2005). The influence of lamination technique on the properties of particleboard. Build. Environ..

[21] Salthammer T., Mentese S., Marutzky R. (2010). Formaldehyde in the Indoor Environment. Chem. Rev..

[22] Zhou B., Wu H.Q., Yang Z.Y. (2018). Effect of thermal treatment temperature on physical and chemical properties of impregnated paper decorated particleboard. Anhui Agric. Sci. Bull..

[23] Liu Y.F., Zhan X.X., Cao J.P., Xv Y.L., Lu M.L., Xin J.M. (2021). Situation and Development Prospects of Surface Decorated Plywood and Blockboard with Paper Impregnated Thermosetting Resins. China Wood-Based Panels.

[24] Zhou Y. (2020). Analyzing on Market Prospect of Surface Decorated Plywood and Blockboard with Impregnated Paper in China. China For. Prod. Ind..

[25] Lu X.Z., Gui C.S., Fang L., Shi X.H., Tang Y.F. (2022). Preparation and performance of impregnated film paper veneered blockboard. J. For. Eng..

[26] Peng L.M. (2018). Analysis of Production and Quality to Surface Decorated Plywood and Blockboard with Paper Impregnated Thermosetting Resins. China Wood-Based Panels.

[27] Zhan X.X., Xie X.Q., Ye J.Y., Zhang X.W., Fang B.J. (2016). Technology of Surface Decorated Plywood and Blockboard Overlaid with Impregnated Paper. China Wood Ind..

[28] Ye J.Y., Shen J.X., Guo B.T. (2018). Analysis of Surface Cracking Within Surface Decorated Plywood and Blockboard with Paper Impregnated Thermosetting Resins. China Wood-Based Panels.

[29] Yuan Q.P., Su C.W., Li N., Yang S., Zeng L.J. (2017). A Multifunctional Impregnated Paper Decorated Wood-Based Panel with Anti-cracking and Its Manufacturing Method. CN Patent.

[30] Dai X.F., Tang Y.F., Gao S.C., Shen Y.F., Chen G.R., Gui C.S., Lu X.C., Shi X.H., Ji D.L., Li X.F. (2020). A Cracking Resistant Ecological Board and Its Production Process. CN Patent.

[31] Shen J.X., Ye J.Y., Shi Q.C., Liu Y.Q. (2019). Research on Manufacturing Technology for Ecological OSB Board of HDF Structure. China Wood-Based Panels.

[32] Fu Z., Zhou F., Gao X., Weng X., Zhou Y. (2020). Assessment of mechanical properties based on the changes of chromatic values in heat treatment wood. Measurement.

[33] Kamke F.A., Lee J.N. (2007). Adhesive penetration in wood—A review. Wood Fiber Sci..

[34] Ye H., Asante B., Schmidt G., Krause A., Zhang Y., Yu Z. (2021). Interfacial bonding properties of the eco-friendly geopolymer-wood composites: Influences of embedded wood depth, wood surface roughness, and moisture conditions. J. Mater. Sci..

