# Supplementary material for: Performance of Impregnated Paper Decorated Blockboard Manufactured Using HDF as Equilibrium Layer
Source: Materials (Basel). 2022 Sep 13;15(18):6342. doi: 10.3390/ma15186342 (PMC9504914; doi:10.3390/ma15186342)
Supplement: Supplementary file 1 [file materials-15-06342-s001.zip › materials-1828815-supplementary.pdf]

Supplementary Material

Performance of Impregnated Paper Decorated Blockboard Manufactured using HDF as Equilibrium Layer

Lu Fang [1,2], Xizhen Lu [2], Xianfeng Mo [1], Xinhao Zhang [2], Chengsheng Gui [3,*]

[1] Co-Innovation Center of Efficient Processing and Utilization of Forest Resources, Nanjing Forestry University, Nanjing 210037, China
[2] College of Furnishings and Industrial Design, Nanjing Forestry University, Nanjing 210037, China
[3] Zhejiang Shenghua Yunfeng Greeneo Co. Ltd., Deqing 313200, Zhejiang, China

* Correspondence: gcs19882006@126.com (C.G.)

File S1: Details of surface bonding strength

At first, cut an annular groove with an inner diameter of 35.7 mm on the surface of the test piece (50 mm x 50 mm). When cutting, it is required to penetrate the decorative material but the depth cut into the wood substrate shall not exceed 0.3 mm. Then bond the special chuck with the specimen using the epoxy resin as adhesive (or hot melt adhesive).

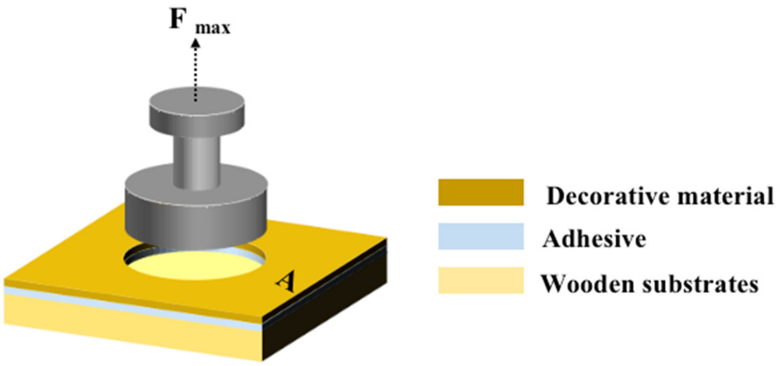

The surface bonding strength was measured using a Multi-Function Mechanical Testing machine (Jinan Shijin Co.Ltd, 10kN), with a crosshead speed of 10 mm min^{-1}. When bonding failure occurred between the decorative material and the substrate, the maximum damage load (Fmax) was output. The surface bonding strength value (∂) was calculated based on the contact area A. (The calculation formula was as follows according to GB/T 17657-2013)

$$\partial = \frac{F_{max}}{A}$$

∂——Surface bonding strength value (MPa)

F_{max}——Maximum load when the surface layer was damaged (N)

A——Bonding area of the specimen to the test tool (1000 mm^2)
